# Connected Vehicles: V2V and V2I Road Weather and Traffic Communication Using Cellular Technologies

**DOI:** 10.3390/s22031142

**Published:** 2022-02-02

**Authors:** Muhammad Naeem Tahir, Pekka Leviäkangas, Marcos Katz

**Affiliations:** 1 Infrastructure & Transport, Faculty of Technology, University of Oulu, FI-90014 Oulu, Finland; pekka.leviakangas@oulu.fi; 2 Centre for Wireless Communications (CWC)-Networks and Systems, University of Oulu, FI-90014 Oulu, Finland; marcos.katz@oulu.fi

**Keywords:** C-ITS, LTE, 5GTN, ITS, V2V, V2I, V2X

## Abstract

There is a continuous need to design and develop wireless technologies to meet the increasing demands for high-speed wireless data transfer to incorporate advanced intelligent transport systems. Different wireless technologies are continuously evolving including short-range and long-range (WiMAX, LTE, and 5G) cellular standards. These emerging technologies can considerably enhance the operational performance of communication between vehicles and road-side infrastructure. This paper analyzes the performance of cellular-based long-term evolution (LTE) and 5GTN (5G Test Network) in pilot field measurements (i.e., vehicle-to-vehicle and vehicle-to-infrastructure) when delivering road weather and traffic information in real-time environments. Measurements were conducted on a test track operated and owned by the Finnish Meteorological Institute (FMI), Finland. The results showed that 5GTN outperformed LTE when exchanging road weather and traffic data messages in V2V and V2I scenarios. This comparison was made by mainly considering bandwidth, throughput, packet loss, and latency. The safety critical messages were transmitted at a transmission frequency of 10 Hz. The performance of both compared technologies (i.e., LTE and 5GTN) fulfilled the minimum requirements of the ITS-Assisted Road weather and traffic platform to offer reliable communication for enhanced road traffic safety. The field measurement results also illustrate the advantage of cellular networks (LTE and 5GTN) with a clear potential to use it heterogeneously in future field tests with short-range protocols, e.g., IEEE 802.11p.

## 1. Introduction

The term intelligent transport system (ITS) is used to demonstrate the design and deployment of information and communication technologies (ICT) in the domain of transportation. ITS offers numerous solutions for road transport by exploiting road weather and traffic data with different communications technologies. Basically, ITS is deployed to improve road traffic safety as well as improve traffic efficiency and reduce ecological impact [1].

A cooperative communication system is one of the key technologies in the framework of ITS. The term “cooperative” signals the collaboration between vehicles and transport infrastructure by using wireless networks. Normally, there are four types of communication in a cooperative intelligent transport system (C-ITS), namely, vehicle-to-vehicle (V2V), vehicle-to-infrastructure (V2I), vehicle-to-pedestrian (V2P), and vehicle-to-network (V2N) [2].

1. V2V Communication: V2V is the direct communication between vehicles’ user equipment (UE);
2. V2I Communication: V2I is the communication between vehicles and road-side infrastructure, i.e., road-side units (RSUs) providing connectivity support to transport infrastructure UE;
3. V2P Communication: V2P is the communication between vehicles and pedestrians UE;
4. V2N Communication: V2N is the wide area cellular communication between vehicles and a cellular infrastructure to assist vehicular traffic functions [2].

**Figure 1:**
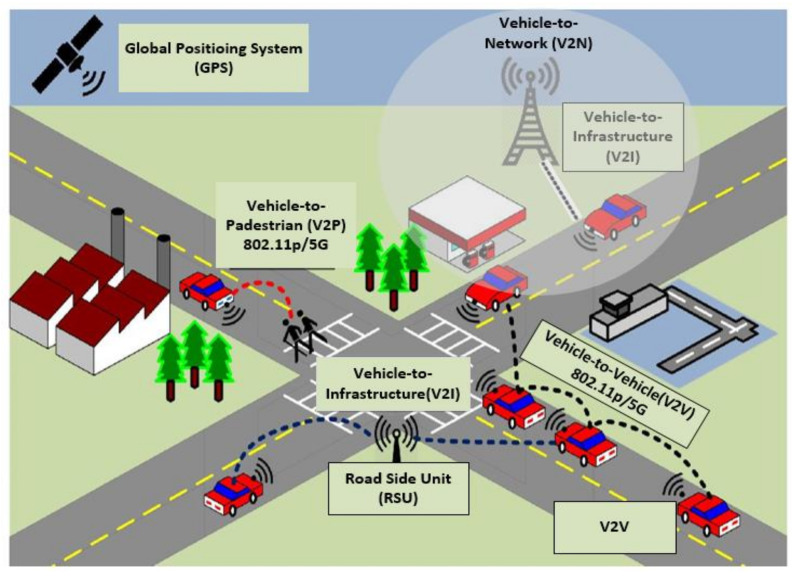
Vehicular communication scenarios in an ITS [3].

The C-ITS communication uses different sensors to assist drivers in particular situations (e.g., maintaining a safe speed and keeping a safe distance from other vehicles, avoiding a probable front-end accident) [3]. These different sensors exchange real-time information between vehicles and transport infrastructure. This information is related to the current road condition and weather updates that might considerably widen the advantages of the aforementioned C-ITS. It also meets the requirements of the different ITS applications related to vehicular communication (V2V, V2I, and V2P) as presented in the Figure 1.

In recent years, a significant work has been conducted for the standardization and allocation of the vehicular communication bandwidth by researchers and scientists globally. Initial success was achieved with the standardization of a dedicated bandwidth for vehicular communication in the United States in 1999. Generally, there are seven 10 MHz channels, and each reserves a 5.9 GHz band for dedicated short-range communication (DSRC). In Europe, the European Telecommunication Standardization Institute (ETSI) standardized the ITS-G5 protocol in the 5.8 GHz frequency band. Both ITS-G5 and DSRC are designed and developed on the basis of IEEE-802.11p [4].

Even though there are several benefits of C-ITS for road traffic safety, there are still some concerns and difficulties related to it. Some of the possible concerns are [5,6] wide-range implementation of C-ITS application; standardization of C-ITS protocols and mutual consensus by several standardization groups; the fundamental challenges of wireless standards (data security problems, multipath propagation, fading issue, etc.).

These issues are important in the development of C-ITS standards in order to avoid accidents and offer much improved road safety.

## 2. Related Work

In recent years, ITS has been the hot topic related to road traffic safety. Many researchers have performed research in the field of ITS focusing on vehicular networking. Some of the prior field measurements related to vehicular networking have also been performed at the FMI Arctic test site in Sodankyla, Finland. In 2013 [7], vehicular networking in operational environments using IEEE-802.11p was presented. IEEE-802.11p is a developed standard based on the IEEE 802.11 standard that adds wireless access to vehicular environments (WAVEs), thus a vehicular communication system. Sukuvaara, Timo, et al. [7] discussed and also proposed a strategy for a deployed system to develop simple use-case scenarios by estimating the system’s efficiency.

In 2019, T. Ojanperä et al. [8] conducted pilot measurements related to the use of road weather services provided by road weather stations (RWS) through Wi-Fi and IEEE 802.11p-based vehicular ad hoc networks (VANETs). The performance of IEEE-802.11p was better in contrast to the Wi-Fi standard. In Reference [9], road weather-related research was performed by considering vehicular networking. The research also discussed a detailed framework of vehicular communication during field measurements by exchanging weather data. In References [10,11], the protocol stacks of ITS in different regions, such as Japan, Europe, and the USA, were reviewed. The study also covered emerging wireless technologies for vehicular networking, i.e., cellular V2X and 5G. This paper presents a comprehensive survey of recent advances in the field of cellular vehicular networks (CVNs). References [12,13] discuss the crucial characteristics of connected vehicular networks including PHY (physical) and MAC (medium access control) as well as routing protocols. The discussion extended to the security aspects of vehicular networking considering link scheduling. References [14,15] conducted a system level comparison of different emerging wireless technologies, i.e., 802.11p and 4G. These studies also performed a comparison by considering real-time field measurements in V2V and V2I scenarios exchanging road weather data.

Due to the rapid demand for high-data-rate ITS applications, the energy consumption model in vehicular networks was discussed in [15] based on combined optimal multi-function game theory and assignment issues. In Reference [16], the author briefly discussed the different factors that restrict the real-time implementation of cellular-based V2X radio resource management design issues.

Recently, FMI conducted a comparative analysis of LTE and 5G in vehicular networking by exchanging real-time weather data. Nevertheless, in this paper, a brief analysis was also conducted according to the collected measurement results in operational environments. This paper focused on the technological and operational attributes of LTE and 5G that are critical for C-ITS services using road weather and friction data. A more detailed description of the features and applications of the LTE and 5G services can be found in [12,13,14,15].

## 3. Aims, Scope, and Structure

The aim of this article was the analysis of the performance of connected vehicles in V2V and V2I scenarios by exchanging road weather and traffic information in cellular networking. The cellular networks used were LTE and 5GTN. These two cellular networks were used to assess the performance of vehicular networking to facilitate traditional VANETs, i.e., IEEE 802.11p/ITS-G5/DSRC.

The Finnish Meteorological Institute has a long history in the design, development, and analysis of network performance by exchanging real-time weather and traffic data using VANET protocols. At the FMI Arctic Research Centre, Sodankyla, a wireless VANET platform has been developed that is able to conduct tests related to weather data and future weather services for automated transport. For pilot use-case scenarios and additional research, a state-of-the-art road-side infrastructure was developed with the combination of an RWS or an RSU to communicate with vehicles by exchanging real-time weather and road traffic data. To analyze and demonstrate the ITS services related to vehicular communication “road weather testbeds” were designed and deployed with advance communication technologies. Likewise, this paper demonstrates the important role of road weather and traffic services by using advanced communication technologies, i.e., cellular technology to provide updated information to vehicles.

This study was a continuation of prior research related to smart mobility and its fundamental and evolving cellular technologies in road transportation. This study mainly focused on vehicular communication by providing new insight and improved results in the domain of vehicular communication. It furthers the previous research work by focusing exclusively on weather and road weather data, which are important aspects of conducting C-ITS operations. Furthermore, the state-of-the-art vehicular networking platform will be beneficial for creating low-latency heterogenous-based technologies (802.11p and the latest cellular technology) [17,18].

The structure of the paper is as follows: Section 4 briefly discusses the C-ITS architecture communication modules using different wireless technologies; Section 5 presents wireless communication technologies, followed by Section 6 that discuses field measurements and test locations. Section 7 briefly analyzes the results followed by their implications and a discussion in Section 8. Finally, in Section 9, the paper is concluded.

## 4. Architecture of a Cooperative Intelligent Transport System

**Figure 2:**
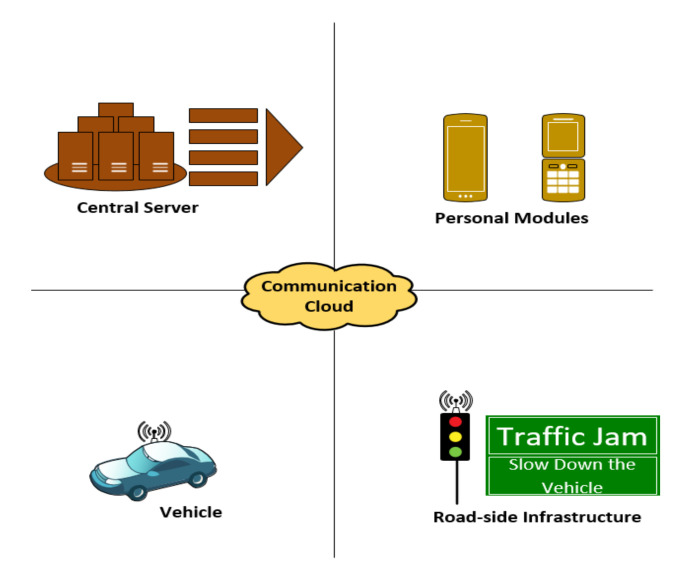
C-ITS architecture communication modules.

In a C-ITS, the design and development of an integrated communications architecture perform crucial roles in the upgrading and larger-scale deployment of a C-ITS architecture. The C-ITS deployment offers different advantages to road users through increased safety, decreased traffic jams, eco-friendly transport systems, etc. These benefits can be achieved by designing of a joint standardized communication framework among different elements in the C-ITS. This C-ITS framework contained four major entities that were composed of different features to form a cooperative intelligent transport system. To create a complete cooperative system, there is no requirement for the availability of all four modules, but a subset of these entities is sufficient. These subsets develop an architecture either by direct communication within the same communication network or indirect communication between different communication networks. The four C-ITS components are illustrated in Figure 2 and are briefly described in the following. For a more detailed description one can refer to [1,7,8].

### 4.1. Central Server

This module is operated and controlled by the traffic administrators in a public authority domain for the management of C-ITS services and applications. The traffic management center is an example of a central serving module that uses road-side units to notify vehicle drivers of current traffic situation, e.g., traffic jams, accidents, and bad weather or blockages. It also recommends alternate itineraries. The central server can collect data from different vehicles and infrastructure and share the updated data with them as well [8].

### 4.2. Personal Module

Basically, this personal module is a roaming machine that can be a smartphone or a navigating unit that can host different ITS applications. The abovementioned devices can also assist C-ITS applications establish a communication link with other transport/road infrastructures and road users [9].

### 4.3. Vehicle Module

The vehicle module is equipped with communication facilities, such as on-board units (OBUs), routers, and embedded computers, to build a network among nearby vehicles and transport or road-side infrastructure. This module has the right to use a vehicle’s CAN bus network to exchange information collected from vehicles as well as to process and transmit data to road-side units or the main server. For a vehicle module, there is no specific defined hardware, because it might be a distinctive hardware unit or several combined units to create a local area network (LAN) within the vehicle [10].

### 4.4. Road-Side Module

The road-side module contains variable message indicators, traffic lights, and other components equipped with communication capabilities. In this way, the road-side module can communicate with other vehicles by transmitting messages or working as a relay offering multi-hop communication in V2I or V2I2V scenarios. Additionally, this module provides a connection with other road-side units and the central server and, thus, forwards the collected data from vehicles [11]. This road-side module also has the ability to create a connection to the internet [12].

### 4.5. Reference Protocol Stack

**Figure 3:**
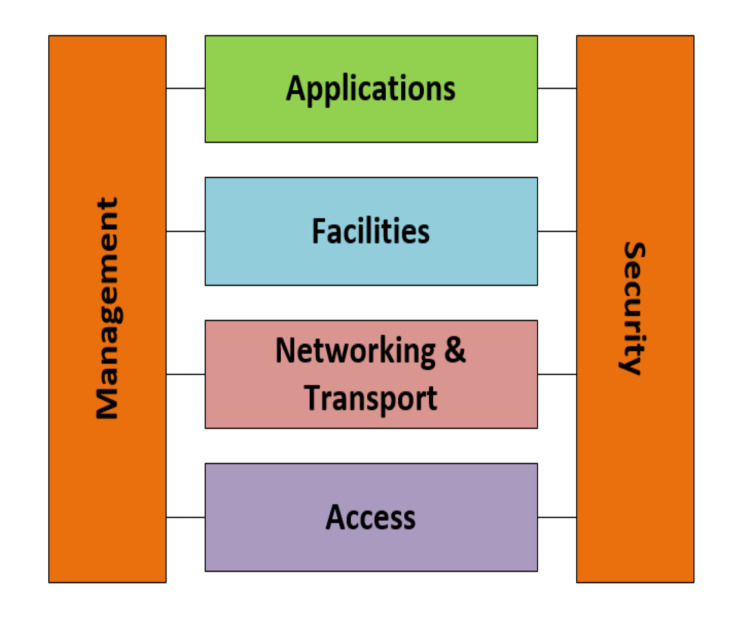
ITS protocol stack.

The aforementioned ITS modules comprise an ITS base station with different ITS specific functions with established devices employing these functions. For C-ITS communication, a protocol stack needs to be considered as illustrated in Figure 3. This protocol stack is built on the on the basis of an ISO/OSI reference model containing four parallel layers and two perpendicular layers that wings the parallel protocol stack [13,14]. The access layer in Figure 3 includes several transmission media and associated protocols for data link and physical layers. The networking and transport layer consists of protocols for information distribution between ITS stations and other network nodes, e.g., network modules in a core network (such as cyberspace). The facilities layer in the ITS protocol stack supports a combination of functionalities to assist applications related to ITS. The application layer is on the top of the ITS protocol stack and realizes the pilot scenarios for road traffic safety, road traffic efficiency, and ITS infotainment. The ITS management layer is used to configure the ITS station for the cross-layer communication between other layers and new ITS-related events. The security layer offers data privacy and security services, e.g., safe and reliable communication between different ITS protocol layers and management of security and identity certificates as well as management of safe platforms by implementing secure gateways, firewalls, etc. [15].

## 5. Cellular Technologies for Vehicular Communication

### 5.1. Long-Term Evolution

In order to understand and formulate the real meaning of the ITS, scientists and researchers are continuously working on the integration of the VANET and cellular technologies together to build a heterogeneous network. This heterogeneous network provides us with new insights to perform in-depth analysis of LTE. Therefore, to make it compatible with the VANET system, the LTE system needs some modification. The initial modification is important for high-mobility vehicles to reduce the latency that directly enhances the quality of service (QOS) by imposing orthogonal frequency division multiplexing (OFDM). To implement this arrangement, the VANET will require authorization to gain access to the cyberspace via existing LTE infrastructure, and this heterogenous system will increase the vehicular communication system’s efficiency. Nonetheless, mobile nodes in large numbers appear as a challenging aspect in this system [16,17,18,19,20]. Hence, the field measurements that were performed and studied in this paper involve a combination of an improved clustering framework, called multi-parallel processing, with multiband OFDM (MP-MBOFDM). This framework aids the reuse of spatial resources, which increases the system’s capability at the cost of additional expenses to manage the high-mobility vehicles [17].

### 5.2. 5G Test Network

The 5th Generation (5G) network is already operational, and it is based on mmWave technology to provide ultra-low latency communication via efficient use of resources. In previous cellular standards, there were issues and limitation factors for vehicular communication. One important issue was the selection of beams that strongly relied on correct localization information and a convoluted transceiver chain, causing unnecessary overhead and network delay. In this paper, 5GTN was used and was built using Nokia’s long-term evolution advance (LTE-A) hardware. The 5GTN is basically deployed for the design and development of 5G applications and services related to vehicular communication. It uses 3.5 GHz by supporting different data rates, varying from 10 to 50 Mbps in the spectrum of 60 MHz in the downlink and uplink correspondingly [18,19,20]. Theoretically, this cellular network may not be the best choice for vehicular communication, but it aids in the development of 5G services and applications and helps to determine issues in current cellular systems. A brief comparison between LTE and 5G is shown below in Table 1.

## 6. Field Measurements and Test Location

**Figure 4:**
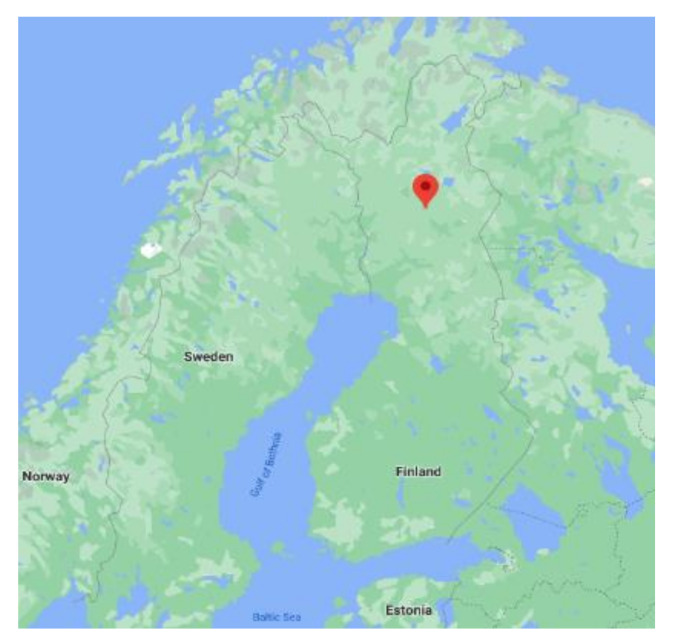
Testing location.

This section briefly describes the field test measurements conducted between June 2020 and August 2020. Using LTE and 5GTN networking, the tests considered V2I and V2V scenarios when exchanging weather and traffic data. The “Sod5G” test track, 1.7 km in length, was used as the location for the tests. The site is north of the Arctic Circle in Sodankylä and hosted by the Finnish Meteorological Institute (Figure 4).

**Figure 5:**
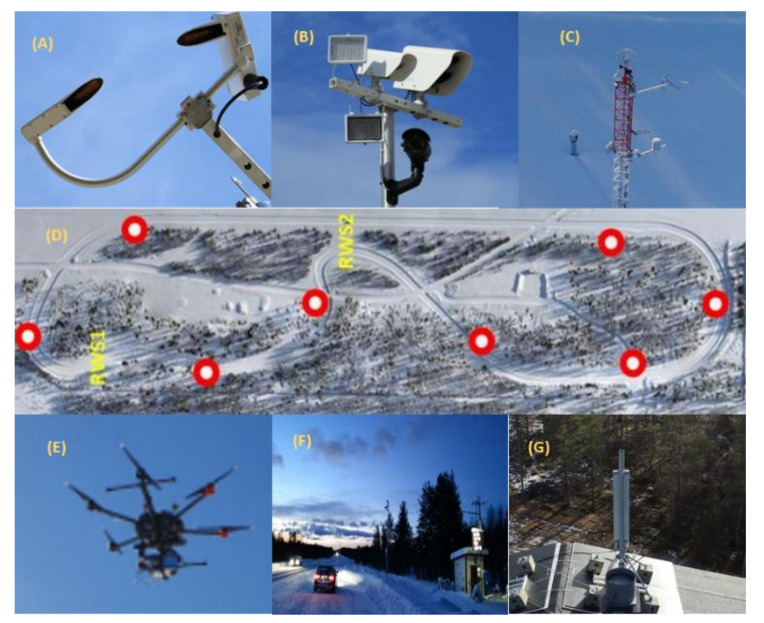
(**A**) Visibility and weather detector; (**B**) surface state sensor; (**C**) road weather station and camera; (**D**) test track; (**E**) test track equipped with IoT sensors (i.e., red spots); (**E**) drone; (**F**) field measurements in a real environment; (**G**) 5GTN road weather stations.

The Sod5G test track is equipped with two RWS, one 5GTN base station, and IoT sensors for weather and traffic data collection as presented in Figure 5.

**Figure 6:**
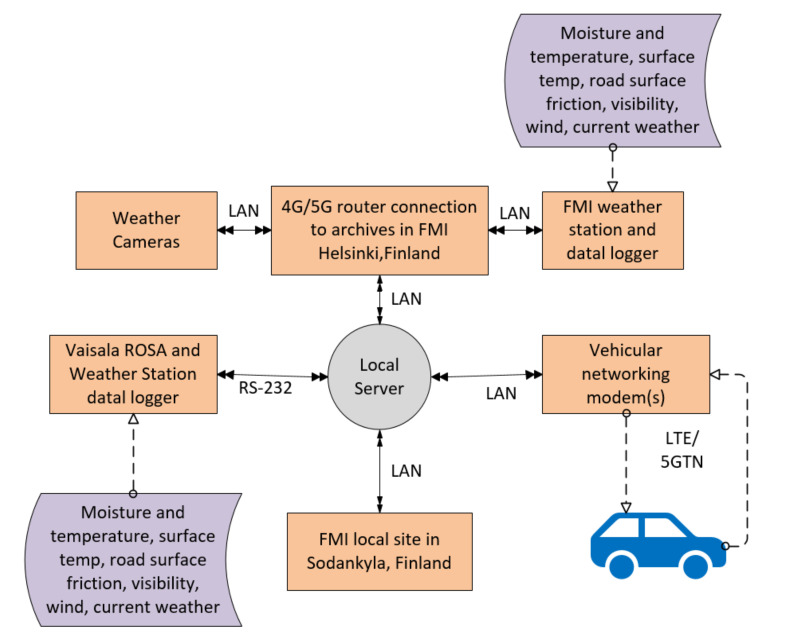
V2V and V2I communication entities.

Figure 6 shows the V2V and V2I communication processes involving different communication entities, e.g., routers and sensors. For the V2V scenarios, two vehicles were used to collect road service data such as collision warnings and friction measurements; temperature sensor data; vehicle telematics data using CAN bus. For the V2I scenarios, a vehicle was used to interact with the two RWS while passing them on the test track.

As presented in the Figure 6 and Table 2, the updated weather data were collected and transmitted from vehicles to other vehicles using LTE and 5GTN. The equipment used for field measurements included Cohda Mk5 and MK6 wireless transceivers, the Python programming language, iPerf, Wireshark, and a smart phone compatible with 5GTN (i.e., Samsung Galaxy S7). For the user Interface (UI), a Sunnit vehicle PC was used. State-of-the-art devices and sensors were used in the field measurements including a Teconer WCM 411 and a Teconer RCM 411 (i.e., vehicles); RWS sensors included the Vaisala PWD-22, DSC-111, Zavio B-7210 FHD camera, and the 2D Ultrasonic Anemometer. The telematics data from the vehicles’ CAN buses were also used for informational content about the vehicles [21,22,23,24]. The used sensors and collected data are presented in Table 2, and some of the IoT sensors (weather sensors, visibility sensors, road state sensors) were installed on the test track with the RWS, which are also illustrated in Figure 5.

During field measurements, the vehicles were equipped with a dual-mode OBU (5GTN and LTE interfaces) moving across the multi-tier networking of 5GTN and LTE. Figure 6 illustrates that the 5GTN and LTE networks provided facilities for vehicles to select the best available network for V2V and V2I communication considering various parameters, e.g., range and power levels. For network selection, scanning was performed by the OBUs installed in the vehicles, and then the best available network sent a beacon message to create a connection between the vehicles and the RWS.

## 7. Results and Analysis

In this section, the field measurement results are discussed for the V2V and V2I scenarios by exchanging weather and road traffic data.

**Figure 7:**
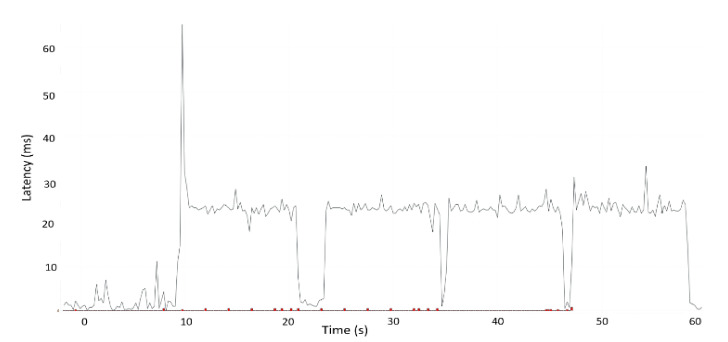
5GTN latency.

**Figure 8:**
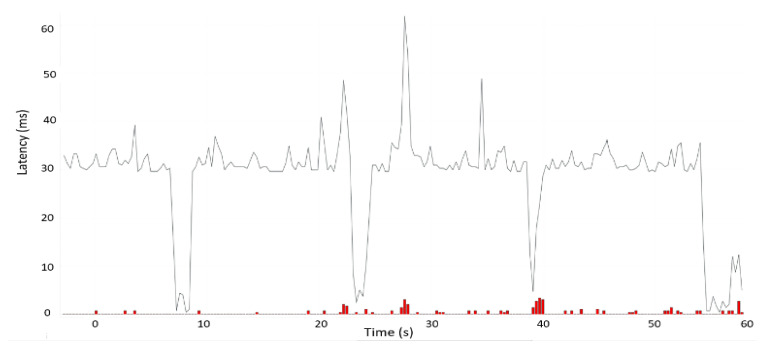
LTE latency.

The Table 3 presents the comparative data of two different cellular networks (i.e., LTE and 5GTN) considering real-time data in similar outdoor environmental conditions. Table 3 illustrates that the performance of the 5GTN was slightly better than the LTE. Figure 7 and Figure 8 illustrate the latency of the LTE and 5GTN in our field measurement results to conduct a comparative analysis between LTE and 5GTN.

The comparative analysis was conducted by considering 10 field measurements conducted on a test track in Sodankyla, Finland. Table 3 together with Figure 7 and Figure 8 reveal that during the start of the test measurements, there were more packet losses which ultimately led to an increase in network latency. However, as a whole, the LTE and 5GTN performances were satisfactory for fulfilling the minimum requirements to deliver the safety messages in the V2V and V2I scenarios (<100 ms) [13,14,15,16,17,18,19,20,21,22,23,24,25,26,27,28].

**Figure 9:**
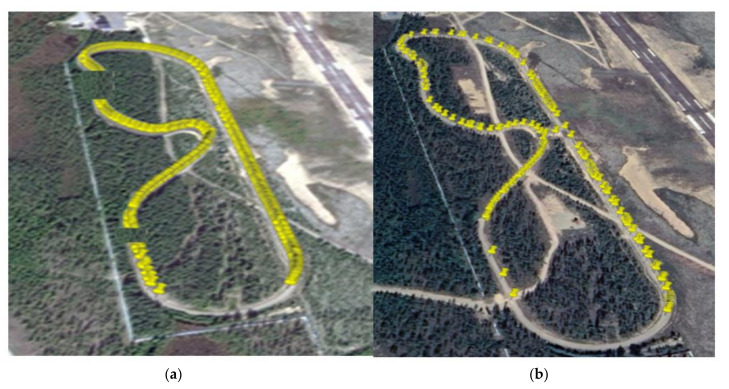
(**a**) Packet capture using the 5GTN. (**b**) Packet capture using the LTE networks.

Figure 9a,b presents UDP packet capture during our field measurements considering the 5GTN and LTE. The yellow spots illustrate the locations where packets were captured during measurements. This packet capture helps to determine the spots where packets are dopped or lost (i.e., packet loss %). It can be noticed in Figure 9a,b that the 5GTN performed better during the measurements. LTE had slightly more packet losses due to the environmental impact while driving the vehicles on the test track. There were neither any obstacles or barrier nor external interferences from other systems and networks.

The variation in the performance of the field measurements depended on the distance and absolute and relative velocities. It can be observed in Figure 9a,b that the 5GTN connectivity had an achievable range of almost 1000 m under an appropriate testing environment. The LTE had a network connectivity range of almost 900 m on our test track during the field measurements.

**Figure 10:**
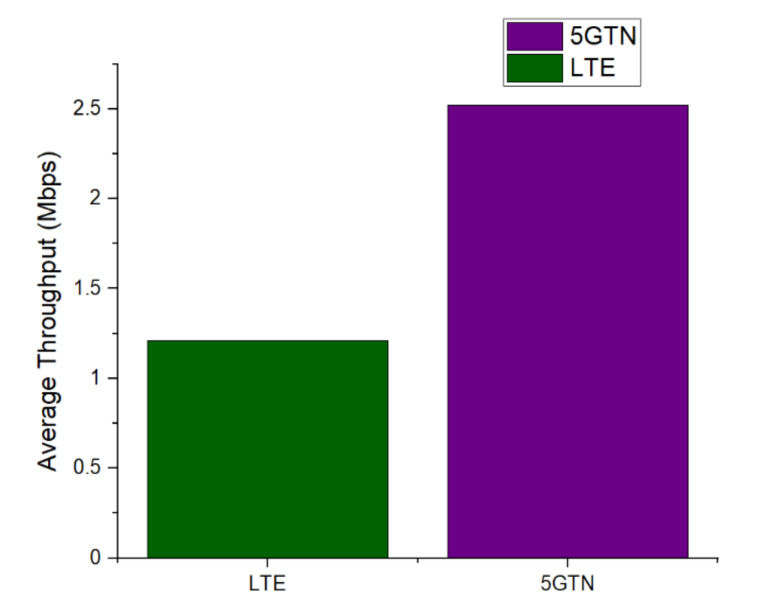
Average throughput comparison between LTE and 5GTN.

Figure 10 shows that the average throughput in the comparison of LTE and 5GTN clearly ranked the performance of 5GTN better. The 5GTN performed well during field measurements since it supports ultra-low latency networking. This average throughput was also affected by the packet loss and latency that made 5GTN better in contrast to LTE. The 5GTN also had a stable throughput as well as bounded packet delay.

## 8. Implications and Discussion

In this section, the implications of the real-time data from the RWS and vehicles are discussed in the context of V2V and V2I. The real-time performance analysis of LTE and 5GTN in vehicular communication provides an opportunity to validate the operational capabilities of the two technologies. The worldwide availability of cellular network makes it possible to exploit the cellular infrastructure together with cellular user equipment for vehicular communication. In the field measurements, the RWS played the role of main infrastructure, supported by sensors and road condition observing vehicles. The results revealed that the performances of the cellular networks were adequate in enhancing road weather data, thereby contributing to road safety by providing enough bandwidth and average throughput. To achieve this road safety, it is aimed to utilize up-to-date road weather and traffic data using LTE and 5GTN [29]. Both networks were able to deliver weather and traffic data efficiently. The LTE-V2X has already been rolled out and is in the deployment phase, but it still has a few issues, e.g., the absence of network availability and the security mechanism need to be considered.

Nevertheless, with the use of cellular technologies, it is necessary to consider the requirements of hardware for vehicular communication, because the equipment for other wireless technologies might be different and they will also need separate solutions. On the other hand, this cellular network would help other short-range networks (IEEE 802.11p/ITS-G5/VLC/Wi-Fi, etc.) to extend the range of vehicular communication by increasing road safety. With the integration of cellular technology using short-range networks, the resulting platform is called a heterogeneous network that leverages the best of both technologies [30,31]. The use of cellular technologies together with short-range protocols would also offer improved load balancing during V2V and V2I, thus attaining low latency with less packet loss (%) in a high-density vehicular network.

As current cellular technologies can only support basic V2X pilot scenarios and lack the support for high mobility and low-latency pilot scenarios, rationally, the use of cellular technology for C-ITS would take many years before it met all the needs of vehicular communication (C-V2X) [32,33,34]. The LTE and 5G networks can complement each other by working together to provide safety in ITS applications. This combined LTE and 5G based C-V2X would be able to offer safety with direct communication for connected and automated vehicles.

## 9. Conclusions

In the domain of intelligent transport systems, vehicular communication is an important aspect for exchanging information between vehicle and transport infrastructure in order to offer considerable road safety. In this paper, a comparison studying the performances of two different cellular networking platforms (i.e., LTE and 5GTN) to support the utilization of weather and road condition data in vehicular communication. The comparison was executed on a test site in Arctic Finland with a weather and road traffic infrastructure equipped with the LTE and 5GTN cellular networks. This comparison of cellular technologies in vehicular environments included average throughput, latency, and network bandwidth. The field measurement results showed that the performance of the 5GTN was better in contrast to the LTE as it was to be expected. Because 5GTN has low latency and packet loss, it ultimately improves the average throughput. Meanwhile, the performance of LTE was also quite reasonable with a slightly low network performance in contrast to 5G.

However, both LTE and 5GTN achieved the application’s requirements (safety, reliability, and low latency) to deliver critical safety messages in V2V and V2I scenarios. It is evident, though, that the launch of 5G is expected to offer even better functionalities and, thus, offer better safety and driver information. These cellular-based vehicular services need effective cooperation between different application service providers (ASPs) to attain the key advantages of cooperative information sharing. In the future, these cellular technologies will be tested and implemented practically on a large scale, and the following phases would require intensifying the pilot scenarios on main roads and highways.

## Figures and Tables

**Table 1:** Technological comparison between LTE and 5G.

Description	LTE	5G
Technology Evolvement	Evolved from 3G to LTE	Evolved from 3G–5G
Frequency Band	1900–2170 MHz	450 MHz to 6 GHz and 24.25–52.6 GHz
Deployment and Scalability	Upgraded from the existing cellular infrastructure	Upgraded from the existing cellular Infrastructure
Network Speed	Up to 10–100 MB	Up to 20 GB
Latency	<10 ms	≤50 ms
Network Resource Usage	200–400 users per cell	100 times greater than LTE 4G
Base Stations	Cell towers	Small cells
OFDM Encoding	20 MHz channels	100–800 MHz channels

**Table 2:** RWS and vehicle data collected during the V2V and V2I scenarios.

Parameters	Sensor	Measurement Height/Depth from the Surface
Current Visibility and Weather Detector (km)	Vaisala PWD-22	10
Dew Point (°C)	PT-100	1.5
Air Temperature (°C)	Vaisala HMP-155 and PT-100	1.8
Wind Speed and direction (m/s)	2D Ultrasonic Anemometer	7
Humidity (%)	HMP-45D	96
Road Surface State (Vehicle and RWS) (m)	Vaisala DSC-111 and DRS-511	5
Pressure (hPa)	2D Ultrasonic Anemometer	1013.3
Infrared Camera (m)	Zavio Full HD B-7210	5
Road Surface Temperature (Vehicle and RWS) (°C)	Vaisala DST-111	−2

**Table 3:** Comparative analysis between 5GTN and LTE.

Measurement Description	LTE	5GTN
Transfer Size (Mbytes)	30.57	29.7
Bandwidth (Mbps)	1.67	3.04
Latency (ms)	38	32
Packet Loss (%)	24	21
Average Packet Size (bytes)	1298	1460
Average Throughput (Mbps)	1.24	2.51
Measurement Time Span (s)	60	60

## Abbreviations

ASP	Application Service Provider
C-V2X	Cooperative Vehicle-to-Everything
CVN	Cellular Vehicular Networks
C-ITS	Cooperative Intelligent Transport System
FMI	Finnish Meteorological Institute
5G	5th Generation
5GTN	5G Test Network
ITS	Intelligent Transport System
LTE	Long Term Evolution
LTE-A	Long Term Evolution-Advance
MAC	Media Access Control
OBU	On-board Unit
OFDM	Orthogonal Frequency Divisiob Multiplexing
QOS	Quality of Service
RSU	Road Side Unit
RWS	Road Weather Station
UE	User Equipment
VANET	Vehicle Adhoc Network
V2I	Vehicle-to-Infrastructure
V2V	Vehicle-to-Vehicle
V2X	Vehicle-to-Everything
VLC	Visible Light Communication
WAVE	Wireless Access in Vehicular Environments

## References

[1] Lytrivis P., Amditis A. (2012). Intelligent Transport Systems: Co-Operative Systems (Vehicular Communications). Wireless Communications and Networks—Recent Advances.

[2] Schlingelhof M., Bétaille D., Bonnifait P., Demaseure K. (2008). Advanced positioning technologies for co-operative systems. IET Intell. Transp. Syst..

[3] Eze E.C., Zhang S., Liu E. Vehicular ad hoc networks (VANETs): Current state, challenges, potentials and way forward. Proceedings of the 2014 20th International Conference on Automation and Computing.

[4] Lianghai J., Liu M., Weinand A., Schotten H.D. (2017). Direct vehicle-to-vehicle communication with infrastructure assistance in 5G network. Ad Hoc Networking Workshop.

[5] Tahir M.N., Katz M. C-ITS Communications using Heterogeneous Wireless Technologies. Proceedings of the IEEE INFOCOM 2021-IEEE Conference on Computer Communications Workshops (INFOCOM WKSHPS).

[6] Santa J., Moragon A., Gomez-Skarmeta A.F. Experimental evaluation of a novel vehicular communication paradigm based on cellular networks. Proceedings of the 2008 IEEE Intelligent Vehicles Symposium.

[7] Sukuvaara T., Maenpaa K., Ylitalo R., Nurmi P., Atlaskin E. Interactive local road weather services through vanet-capable road weather station. Proceedings of the 20th ITS World Congress ITS Japan.

[8] Sukuvaara T., Mäenpää K., Ylitalo R. (2016). Vehicular-networking- and road-weather-related research in Sodankylä. Geosci. Instrum. Methods Data Syst..

[9] Ojanperä T., Kutila M., Pyykönen P., Scholliers J., Sukuvaara T., Mäenpää K., Huuskonen O. Development and Piloting of Novel 5G-Enabled Road Safety Services. Proceedings of the 2019 IEEE Wireless Communications and Networking Conference Workshop (WCNCW).

[10] Singh P.K., Nandi S.K., Nandi S. (2019). A tutorial survey on vehicular communication state of the art, and future research directions. Veh. Commun..

[11] Tahir M.N., Mäenpää K., Sukuvaara T., Leviäkangas P. (2021). Deployment and Analysis of Cooperative Intelligent Transport System Pilot Service Alerts in Real Environment. IEEE Open J. Intell. Transp. Syst..

[12] Rawat D.B., Yan G. (2010). Infrastructures in vehicular communications: Status, challenges and perspectives. Advances in Vehicular Ad-Hoc Networks: Developments and Challenges.

[13] Ahmed E., Gharavi H. (2018). Cooperative Vehicular Networking: A Survey. Proceedings of the IEEE Transactions on Intelligent Transportation Systems.

[14] Wang X., Mao S., Gong M.X. (2017). An overview of 3GPP cellular vehicle-to-everything standards. GetMobile Mob. Comput. Commun..

[15] Olia A., Abdelgawad H., Abdulhai B., Razavi S.N. (2016). Assessing the potential impacts of connected vehicles: Mobility, environmental, and safety perspectives. J. Intell. Transp. Syst..

[16] Tahir M.N., Katz M. (2021). Heterogeneous (ITS-G5 and 5G) vehicular pilot road weather service platform in a realistic operational environment. Sensors.

[17] Sheeba H., Gaur D., Kumar Shukla V. Impact of Emerging Technologies on Future Mobility in Smart Cities by 2030. Proceedings of the 2021 9th International Conference on Reliability, Infocom Technologies and Optimization (Trends and Future Directions) (ICRITO).

[18] Loussaief F., Marouane H., Koubaa H., Zarai F. (2020). Radio resource management for vehicular communication via cellular device to device links: Review and challenges. Telecommun. Syst..

[19] Ren Y., Liu F., Liu Z., Wang C., Ji Y. (2015). Power control in D2D-based vehicular communication networks. IEEE Trans. Veh. Technol..

[20] Mintsis E., Vlahogianni E.I., Ozkul S. (2021). Enhanced speed advice for connected vehicles in the proximity of signalized intersections. Eur. Transp. Res. Rev..

[21] Tahir M.N., Katz M., Pouttu A. VANET (ITS-G5 & 5G Test Network) with Drone-assisted Communication Using Road Weather Information. Proceedings of the 2021 17th International Conference on Wireless and Mobile Computing, Networking and Communications (WiMob).

[22] Tahir M.N., Katz M., Rashid U. (2021). Analysis of collaborative wireless vehicular technologies under realistic conditions. J. Eng..

[23] Paruchuri V. Inter-vehicular communications: Security and reliability issues. Proceedings of the ICTC 2011.

[24] Tahir M.N., Rashid U. Demo: Intelligent Transport System (ITS) Assisted Road Weather & Traffic Services. Proceedings of the 2020 IEEE Vehicular Networking Conference (VNC).

[25] Tahir M.N., Katz M., Rashid U. Analysis of VANET Wireless Networking Technologies in Realistic Environments. Proceedings of the 2021 IEEE Radio and Wireless Symposium (RWS).

[26] Cheng H.T., Shan H., Zhuang W. (2011). Infotainment and road safety service support in vehicular networking: From a communication perspective. Mech. Syst. Signal Processing.

[27] Araniti G., Campolo C., Condoluci M., Iera A., Molinaro A. (2013). LTE for vehicular networking: A survey. IEEE Commun. Mag..

[28] Astarita V., Guido G., Giofrè V.P. (2014). Co-operative ITS: Smartphone based measurement systems for road safety assessment. Procedia Comput. Sci..

[29] Tahir M.N., Katz M., Hamid U.Z.A., Al-Turjman F. (2021). ITS Performance Evaluation in Direct Short-Range Communication (IEEE 802.11p) and Cellular Network (5G) (TCP vs. UDP). Towards Connected and Autonomous Vehicle Highways.

[30] Schroten A., van Grinsven A., Tol E., Leestemaker L., Schackmann P.P.M., Vonk Noordegraaf D.M., van Meijeren J.C., Kalisvaart S. (2020). The Impact of Emerging Technologies on the Transport System.

[31] Filippi A., Moerman K., Daalderop G., Alexander P.D., Schober F., Pfliegl W. Ready to Roll: Why 802.11 p Beats LTE and 5G for V2x. NXP Semiconductors, Cohda Wireless and Siemens White. https://assets.new.siemens.com/siemens/assets/api/uuid:ab5935c545ee430a94910921b8ec75f3c17bab6c/its-g5-ready-to-roll-en.pdf.

[32] Tahir M.N., Katz M. (2021). Performance evaluation of IEEE 802.11 p, LTE and 5G in connected vehicles for cooperative awareness. Eng. Rep..

[33] Kaiwartya O., Abdullah A.H., Cao Y., Altameem A., Prasad M., Lin C.-T., Liu X. (2016). Internet of Vehicles: Motivation, Layered Architecture, Network Model, Challenges, and Future Aspects. IEEE Access.

[34] Tahir M.N., Katz M., Javed Z. Poster: Connected Vehicles using Short-range (Wi-Fi & IEEE 802.11 p) and Long-range Cellular Networks (LTE & 5G). Proceedings of the 2021 IEEE 29th International Conference on Network Protocols (ICNP).

